# Slowed Biogeochemical Cycling in Sub-arctic Birch Forest Linked to Reduced Mycorrhizal Growth and Community Change after a Defoliation Event

**DOI:** 10.1007/s10021-016-0026-7

**Published:** 2016-08-25

**Authors:** Thomas C. Parker, Jesse Sadowsky, Haley Dunleavy, Jens-Arne Subke, Serita D. Frey, Philip A. Wookey

**Affiliations:** 1 Biological and Environmental Sciences, School of Natural Sciences, University of Stirling, Stirling, FK9 4LA UK; 2 Department of Animal and Plant Sciences, University of Sheffield, Alfred Denny Building, Sheffield, S10 2TN UK; 3 Department of Natural Resources and the Environment, University of New Hampshire, Durham, New Hampshire USA; 4 Environmental Sciences, School of Life Sciences, Heriot-Watt University, Edinburgh, EH14 4AS UK; 5 Present Address: The Ecosystems Center, Marine Biological Laboratory, Woods Hole, Massachusetts 02543 USA

**Keywords:** defoliation, nitrogen, carbon, birch forest, sub-arctic, ectomycorrhizal fungi, community change

## Abstract

Sub-arctic birch forests (*Betula pubescens* Ehrh. ssp. *czerepanovii*) periodically suffer large-scale defoliation events caused by the caterpillars of the geometrid moths *Epirrita autumnata* and *Operophtera brumata*. Despite their obvious influence on ecosystem primary productivity, little is known about how the associated reduction in belowground C allocation affects soil processes. We quantified the soil response following a natural defoliation event in sub-arctic Sweden by measuring soil respiration, nitrogen availability and ectomycorrhizal fungi (EMF) hyphal production and root tip community composition. There was a reduction in soil respiration and an accumulation of soil inorganic N in defoliated plots, symptomatic of a slowdown of soil processes. This coincided with a reduction of EMF hyphal production and a shift in the EMF community to lower autotrophic C-demanding lineages (for example, /russula-lactarius). We show that microbial and nutrient cycling processes shift to a slower, less C-demanding state in response to canopy defoliation. We speculate that, amongst other factors, a reduction in the potential of EMF biomass to immobilise excess mineral nitrogen resulted in its build-up in the soil. These defoliation events are becoming more geographically widespread with climate warming, and could result in a fundamental shift in sub-arctic ecosystem processes and properties. EMF fungi may be important in mediating the response of soil cycles to defoliation and their role merits further investigation.

**Electronic supplementary material:**

The online version of this article (doi:10.1007/s10021-016-0026-7) contains supplementary material, which is available to authorized users.

## Introduction

Mountain birch trees (*B.* *pubescens* Ehrh. ssp. *czerepanovii* (Orlova) Hämet Ahti) comprise the dominant treeline forests in most of the northern Fennoscandia (Tømmervik and others 2009; Hofgaard and others 2013). This forest is responsive to climate change, amongst other important drivers such as changes in reindeer management (Tømmervik and others 2009; Van Bogaert and others 2011). The birch treeline has been observed to have advanced both in latitude in the last century (Hofgaard and others 2013) and in elevation in the last 34 years (Rundqvist and others 2011).

Insect outbreaks are important controls over productivity in temperate, boreal (Hicke and others 2012) and sub-arctic ecosystems (Bjerke and others 2014). Cyclical outbreaks of the defoliating Autumnal Moth (*Epirrita autumnata*) and the Winter Moth (*Operophtera brumata*) are common and widespread across the mountain birch forests of Northern Scandinavia (Jepsen and others 2008). These outbreaks occur in waves across the Scandes mountains, with an approximate 10-year frequency (Tenow and others 2007), causing considerable damage to the canopy of *B.* *pubescens* forests (Jepsen and others 2013) and contributing towards large decreases in forest productivity (Bjerke and others 2014). It is not clear whether the frequency of these outbreaks is increasing with climate change, but the area of forest affected has increased by about 5° East in longitude, into the colder continent, and about 2° North in latitude over the last century (Jepsen and others 2008). This is thought to be due to a warming of winter climate, allowing over-winter survival of eggs in areas that were previously too cold (Jepsen and others 2008). With the distribution and severity of defoliator insect outbreaks expected to increase further with climate change (Bale and others 2002; Jepsen and others 2008, 2011), it is important to understand how the mountain birch forest ecosystem responds to such disturbance. Furthermore, the potential links between forest dynamics and both net carbon fluxes and surface energy budget underscore the need to investigate the role of herbivores as modulators of climate change impacts in these ecosystems (Moore and others 2013).

Sub-arctic forests are known to influence soil carbon (C) fluxes and metabolism by allocating recently assimilated C belowground, stimulating the decomposition of soil organic C and the release of nutrients (Hartley and others 2012). Belowground transfer of labile C from trees to the rhizosphere drives microbial activity, soil respiration (Högberg and others 2001) and N immobilisation (Kaiser and others 2011; Näsholm and others 2013a). Because defoliation reduces the ability of sub-arctic birch forests to fix C (Heliasz and others 2011), it is expected that defoliation events strongly reduce C inputs to the rhizosphere and slow biogeochemical cycles.

In addition to altering C allocation patterns, defoliation events in sub-arctic ecosystems accelerate nitrogen (N) inputs into the soil via direct frass addition, thereby potentially altering N cycling (Kaukonen and others 2013). Nitrogen immobilisation in the soil is known to be driven by autotrophic C inputs (Kaiser and others 2011) and belowground C allocation is positively correlated with forest productivity (Litton and others 2007). In productive temperate ecosystems, the N cycle responds quickly to N additions from caterpillar frass through redistribution of N into microbial communities (Lovett and Ruesink 1995) or re-assimilation by the affected trees (Russell and others 2004; Frost and Hunter 2007). In a less productive ecosystem, such as the sub-arctic, reduced C supply to the rhizosphere alongside frass addition may result in an accumulation of ‘available’ N in the soil, as has previously been observed (Kaukonen and others 2013), and akin to an N-saturated ecosystem (Aber 1992).

Ectomycorrhizal fungi (EMF) are a major recipient of autotrophic C in forest ecosystems, with up to 20% of plant C allocated belowground transferred to the EMF community (Högberg and others 2001; Hobbie 2006). This C supply allows EMF species to maintain dominance in the organic horizon of boreal forest soils over free-living fungi (Lindahl and others 2007), but this dominance can be disrupted by cutting the autotrophic C supply (Lindahl and others 2010). Therefore, any reduction in C inputs belowground may translate directly to a reduction in C supply to the mycorrhizosphere (Gehring and others 1997; Högberg and others 2001). EMF fungi have a large capacity to immobilise soil N in their biomass (Näsholm and others 2013a), and a reduction in EMF growth due to defoliation could significantly reduce their capacity to immobilise the free N that becomes available in such events (Kaukonen and others 2013).

When trees are defoliated, it has been speculated that EMF fungi with lower C demand from their autotrophic host hold a competitive advantage over species that require a larger C investment (Saikkonen and others 1999; Markkola and others 2004). Widespread defoliation should therefore drive a change in EMF community composition, selecting for less C-demanding EMF taxa. A shift to exploration types (ET (Agerer 2001)), which produce less hyphal biomass, could have significant feedbacks for C and N cycling in the soil.

Carbon allocation to EMF can stimulate N immobilisation in low N environments by increasing uptake and incorporation into hyphal biomass (Näsholm and others 2013a). It has been shown that efficient uptake of N by EMFs further intensifies N limitation in the soil, thereby maintaining their host’s reliance on them and competitively excluding non-EMF plants (Näsholm and others 2013a). This cycle could be broken by disturbance such as defoliation, where autotrophic C supply to EMF is reduced (Kuikka and others 2003) and mineral N becomes more readily available (Kaukonen and others 2013). High mineral N concentrations in the soil after defoliation events may be due in part to reduced immobilisation by EMF as a result of reduced C supply.

A mountain birch forest (*B.* *pubescens* ssp. *czerepanovii*) in sub-arctic Sweden was defoliated by a joint outbreak of the winter and autumnal moths (*O.* *brumata* and *E.* *autumnata*) in early summer of 2013 after an outbreak the previous year. This gave us the opportunity to measure the belowground response of this ecosystem to the associated reduction in autotrophic C supply. In particular, we address the following research aims: 1. to measure belowground C and N cycling in response to defoliation of the *Betula* canopy; 2. to compare hyphal growth and root tip community composition of ectomycorrhizal fungi in defoliated and non-defoliated plots; and 3. to understand better how changes (if any) in soil C and N cycling link to ectomycorrhizal community composition and growth in a sub-arctic forest ecosystem.

## Materials and Methods

### Study Site

**Figure 1:**
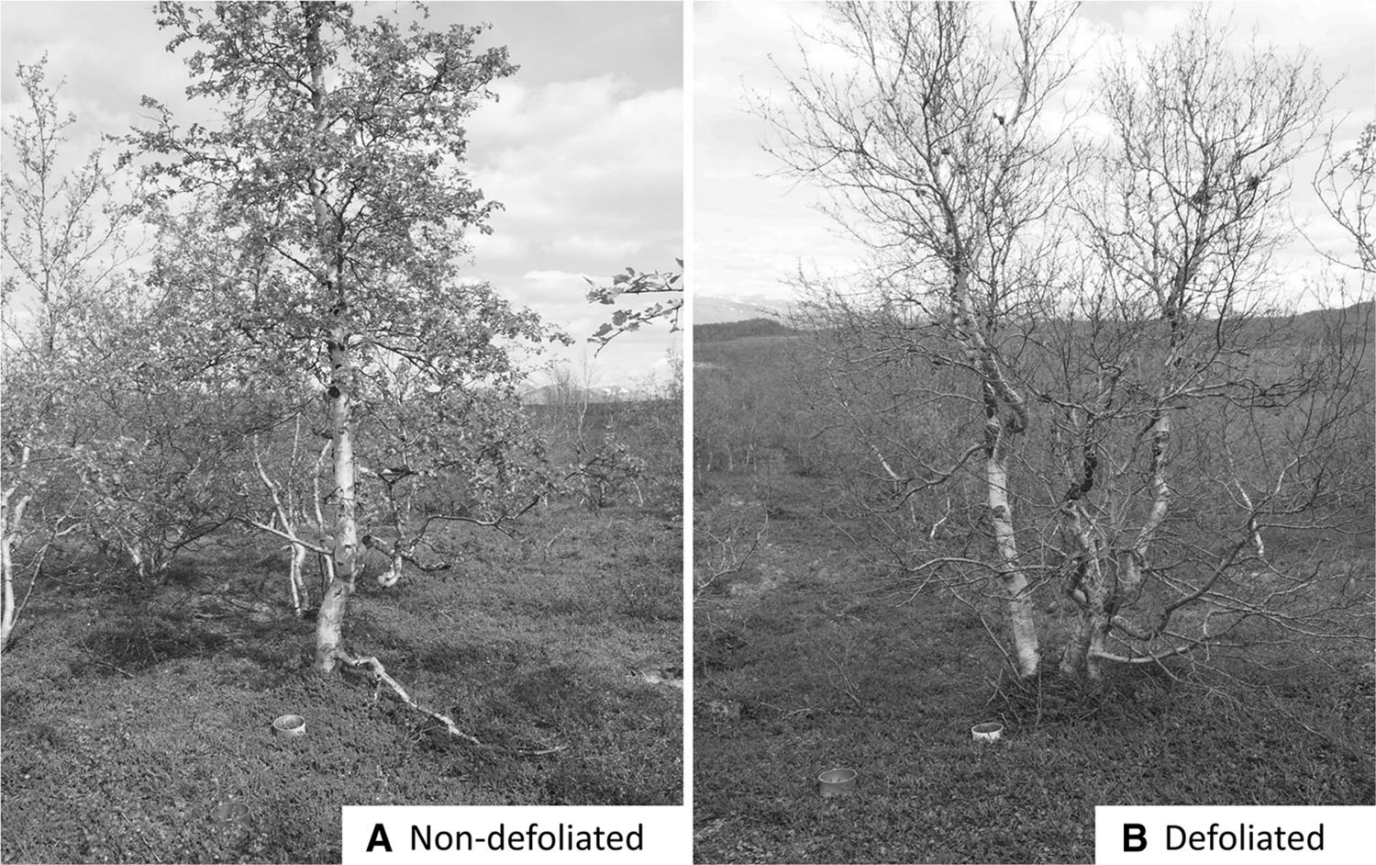
Two *Betula pubescens* study trees assigned to **A** “non-defoliated” and **B** “defoliated” categories based on the extent of defoliation by *Operophtera brumata* and/or *Epirrita autumnata* on 19th June 2013. Collars to measure soil respiration can be seen at 50 and 150 cm from the base of each tree.

Study sites were established in the treeline birch forest near Abisko, Sweden (~68°18′N, 18°49′E). The forest comprises mountain birch (*B.* *pubescens* Ehrh. ssp. *czerepanovii* (Orlova) Hämet Ahti) with a dominantly ericaceous understorey of *Empetrum hermaphroditum, Vaccinium myrtillus, Vaccinium vitis-idaea, Vaccinium uliginosum* and some shrubs including *Betula nana, Salix* spp. and *Juniperus communis.* The soil is a thin spodosol developed over glacial till and bedrock typically of hard shale, with a thin (<5 cm) O horizon. Soil pH of the organic horizon is 4.5 ± 0.1 (mean ± standard error) (Parker and others 2015). Further details on soil properties can be found in Sjögersten and Wookey (2002) and Hartley and others (2010). The forest defoliation event by *O.* *brumata* and *E.* *autumnata* began in May 2013 as budburst occurred across the forest, although the exact timing was highly dependent on local microclimates. The trees were at their maximum extent of defoliation and caterpillars were no longer present in the trees by 19th June 2013 (Figure 1). There was also widespread defoliation due to an outbreak by the same species the previous summer, which is unusual, but this earlier outbreak was not documented in detail in this study area.

## Study Design

Based on the visual extent of defoliation, replicate trees across a forest stand were selected as either non-defoliated (at least 95% of leaves remaining) or defoliated (max. 5% of leaves remaining), with *n* = 5 per group (Figure 1). The trees were selected in geographical pairs over forested areas of approximately 19,000 m^2^, and paired individuals were, on average, 18 m apart and 58 m from the closest other pair. In addition to the defoliated and non-defoliated plots in one *B.* *pubescens* forest stand, other forest stands were sampled to assess defoliation impacts at the landscape scale (LS). LS plots (24 in total, consisting of all trees within a 5 m radius of a central point) were distributed across multiple stands of mountain birch which showed contrasting degrees of defoliation (11.5–96.1% canopy defoliated in July 2013) over a 2 km^2^ area. To quantify defoliation in each plot, every *B.* *pubescens* individual was estimated for percentage of leaves remaining, which was then converted to percentage defoliated (assuming a full canopy prior to defoliation). Defoliation values are means of independent estimates by two observers. Respiration and ion exchange measurements were taken at the defoliated and non-defoliated paired plots; ion exchange, EMF ingrowth and EMF root tips assessments were taken at the LS plots.

### Respiration

At defoliated and non-defoliated plots, two PVC collars (15 cm diameter × 7 cm high) were fixed to the soil surface at 50 and 150 cm from the base of each tree (Figure 1). To avoid disturbance to the rhizosphere, collars were not pushed into the soil, but were sealed to the soil using non-setting putty (Plumber’s Mait^®^, Bostik Ltd, Stafford, UK). Collar locations were selected to have low plant cover within the collar (live understorey <20%, for both defoliated and non-defoliated plots) and therefore capture soil respiration best while causing minimal extra disturbance to the study system over the short period of time available for measurement. A good seal with the ground was confirmed as all respiration measurements showed a linear increase in CO_2_ concentration over time (over 90 s). Live understorey plant cover was low (c 20%) and did not differ between defoliated and non-defoliated collars in the plots.

Soil respiration measurements (which included both microbial and plant components, including very low shoot components) were made with a portable EGM-4 infrared gas analyser with a darkened CPY-2 chamber (PP Systems International, Amesbury, MA, USA). CO_2_ flux was measured five times at each collar through June and July 2013, after the defoliation event, and then twice in September 2013. Follow-up respiration measurements were made in June and July 2014 (one measurement each month). In 2014, ‘defoliated’ trees did not re-grow their canopy, instead investing in new shoots at their base. Respiration rates were calculated as the product of a linear function of [CO_2_] increase over a period of 90 s within the closed system. Tests with longer measurement periods showed no improvement of fit. All collars were measured within a two-hour period between 09:00 and 16:00 h local time.

### Soil Inorganic Nitrogen Availability

Anion and cation exchange membranes (2.5 × 5 cm; Resintech, West Berlin, NJ, USA) were deployed in pairs approximately 5 cm apart to measure soil inorganic N availability in summer (10th–24th July 2013) and autumn (6th–20th September 2013). Membranes were regenerated in 0.5 M HCl for one hour before being neutralised in 0.5 M NaCO_3_ for five hours, replacing the NaCO_3_ every hour. The membranes were inserted vertically into the soil surface (0–5 cm) at the centre of all *LS* plots. Care was taken to select soils with no moss species (for example, *Pleurozium schreberi*) associated with N-fixing cyanobacteria (DeLuca and others 2002) to avoid measuring leached N from this potential source. A knife was used to create a vertical incision in the soil into which the membrane was carefully inserted. The soil was then pushed together to ensure good contact and membranes were left *in situ* for 14 days. Membranes were deployed in the same manner at the paired defoliated and non-defoliated trees, in between the soil respiration collars at 0.75 m from the base of the tree. After collection, adhering soil particles were gently brushed away, after which the membranes were rinsed with deionised water. Membranes were stored at 3°C for 18 days before extraction (100 rpm for 60 min in 35 ml 2 M KCl (Qian and Schoenau 2002)). Extractable NH_4_^+^ and NO_3_^−^ was quantified using flow injection analysis (FIAflow2, Burkard Scientific, Uxbridge, UK). Control strips (*n* = 10 per season) were taken into the field on the day of strip insertion but not placed in the field. They were taken back to the lab and stored at 3°C until field samples were analysed, at which point they were processed in the same way. The mean amount of NH_4_^+^ or NO_3_^−^ adsorbed to control strips in each season was subtracted from field samples as an analytical blank.

### Ectomycorrhizal Hyphal Production

Nylon mesh bags (5 × 4 cm; 37 *µ*m mesh size), which allowed ingrowth of hyphae, anticipated to be primarily of EMF fungi (analysis of community DNA shows c 80% EMF (Wallander and others 2013)) but not roots (Wallander and others 2001, 2013), were filled with 25 g of sand from the shore of Lake Torneträsk (68°21N, 18°49E). No plants were present aboveground within 1 m of the sand collection point. Sand was sieved to between 0.125 and 1 mm, rinsed under a flow of water for 1 min then microwaved (800 W) for 12 min, reaching a temperature of 98°C. This process was repeated and the sand was rinsed a final time before drying for 48 h at 80°C. The sand-filled bags were inserted at the LS plots within 0.5 m of the ion exchange membranes at the centre of the plots. Bags were inserted at the interface between organic and mineral horizons where mycorrhizal activity is at its highest (Lindahl and others 2007); in this case, each bag was placed between 2 and 7 cm depth. The bags were left in the field for 92 days between 16th June and 16th September 2013. This was deemed to be an appropriate time period as C allocation belowground continues into September in this ecosystem (Sloan and others 2015; Blume-Werry and others 2016). At collection, the sand was removed from the bags and freeze dried using a Modulyo^®^ freeze drier (Thermo Fisher Scientific, Waltham, MA, USA) for 72 h within 6 h of recovery.

Sand (1 g) from each bag was sonicated for 10 min in 30 ml deionised water to disassociate the fungal hyphae from the sand particles. A 4 ml aliquot of the water-hyphae suspension was filtered onto a nitrate cellulose filter paper (0.45 *µ*m pore size) and fungal material was stained with trypan blue (following Quirk and others (2012)). Hyphal length was estimated under ×200 magnification (Primo Star, Zeiss, Oberkochen, Germany) using the line intersect method (Brundrett and others 1994). This was repeated on duplicate samples for each mesh bag, a mean of which was taken as the final measurement.

### Ectomycorrhizal (EMF) Community Composition

Root tips of non-defoliated and defoliated trees were collected and analysed to identify the EMF taxa colonising the roots. Samples were taken on 7th July 2013. Five pairs of defoliated and non-defoliated trees were selected to sample EMF root tips. Paired trees located within 5–10 m of each other were designated ‘defoliated’ or ‘non-defoliated’ based upon the percentage of leaves remaining, where about 0 to 15% were designated as ‘defoliated’ and about 85 to 100% were designated as ‘non-defoliated’. Three of these paired plots were selected from the *LS* plots and two from two additional mountain birch stands. At each tree, organic horizon soil was collected as 5.7 cm diameter cores to a depth of 4 cm. Roots within each core were rinsed of adhering soil particles under a stream of tap water on a 1 mm-mesh sieve. From each sample, 48 individual EMF-infected root tips were excised from larger root fragments under a stereomicroscope and stored in tap water at 4°C for up to 14 days prior to DNA extraction. EMF root tips were assumed to be of *B.* *pubescens* because the understorey is primarily ericaceous (ericoid mycorrhizal), with other EMF species present at these plots (*B.* *nana*) typically having very low cover (8 ± 2% (Parker and others 2015)).

To characterise EMF communities, fungal DNA of a random subset of 16 EMF root tips from a pool of 48 per plot was sequenced. Single root tip DNA was extracted with the Extract-N-Amp kit (Sigma, USA), according to Avis and others (2003). Fungal DNA was amplified using polymerase chain reaction (PCR) with the ITS1F-ITS4 primer set (White and others 1990; Gardes and Bruns 1993) at 0.35-*µ*M concentration in GoTaq G2 Master Mix (Promega, USA). The PCR consisted of a 3-min hot start at 95°C, 35 cycles of 30 s at 95°C, 45 s at 60°C and 90 s at 72°C, and a final cycle of 5 min at 72°C. Negative controls (diethylpyrocarbonate-treated water) were included in each PCR run. PCR products were run in 0.05% ethidium bromide 1.5% agarose (w/v) gels and photographed under UV light to confirm single PCR amplicons. After primers and unincorporated nucleotides were removed using ExoSAP (Affymetrix, Cleveland, OH, USA), as described by Kennedy and Hill (2010), amplicons were sequenced with the ITS4 primer on a 3730XL Applied Biosystems sequencer by Macrogen Corp. (Rockville, MD, USA). Sequence chromatograms were edited in FinchTV 1.4.0 (Geospiza, Seattle, WA, USA) or 4peaks 1.7 (http://nucleobytes.com/index.php/4peaks) to eliminate spurious base calls on the flanking ends of sequences.

Fungal sequences were assigned to one of approximately 80 described EMF lineages (Tedersoo and Smith 2013) or to the genus level for non-EMF fungi. Lineages are designated by a ′/′ (slash) followed by the dominant genus, genera, or higher level taxon, for example, /cenococcum, /tomentella-thelephora and /atheliales3. We then computed richness, evenness and diversity (Shannon and Simpson indices) of EMF lineages according to McCune and Grace (2002). It is important to note that only roots with fungal infection were taken forward for DNA analysis; therefore, diversity indices analysed here do not take into account change in overall abundance of ECMs, only the fungi present on roots.

## Statistical Analyses

Respiration data in 2013 and 2014 were analysed separately using a repeated measures two-way ANOVA following a linear mixed effects model with distance from tree and defoliation status (defoliated or non-defoliated) as categorical main effects. Data were square root transformed to meet the assumptions of the parametric analyses. A linear model was used to analyse the relationship between NH_4_^+^ adsorbed to cation exchange membranes and the defoliation extent of the *B.* *pubescens* on the LS plots once the response variable was natural-log transformed. The relationship between defoliation extent and EMF ingrowth was analysed using a linear model. Once again, a natural-log transformation of the response variable made the data appropriate for parametric analysis. One outlying point was removed from the EMF ingrowth analysis because it had a disproportionate effect on the statistical model, violating the underlying assumptions (Cook’s distance > 0.5). A conservative Bonferroni test on its residual confirmed that this was indeed a statistical outlier (*P* = 0.0058) (Kutner and others 2005).

We compared richness, evenness and diversity (both Shannon’s and Simpson’s indices) of EMF lineages present on root tips between defoliated and non-defoliated trees with paired *t* tests. To test a null hypothesis that EMF communities were not affected by defoliation, we used the non-parametric blocked multi-response permutation procedure in PC-ORD (McCune and Mefford 2011). The raw data matrix included counts of EMF fungal lineages and non-EMF fungi in defoliated and non-defoliated plots (*n* = 5). We considered plots as random blocks, performed within-block median averaging, and used distance function commensuration to give equal weighting to variables in the calculated Euclidian distance matrix (McCune and Grace 2002). EMF lineages were grouped into ET (Agerer 2001) based on their identification at lineage level. Differences in relative abundance of different ETs between defoliated and non-defoliated plots were analysed by Holm–Tukey multiple comparisons after a two-way ANOVA with defoliation status and ET as fixed effects.

## Results

There was a marginally significant (*P* = 0.058) positive linear relationship between the density of trees (*x*) and extent of defoliation (*y*) of each tree, as follows: *y* = 26.6 + 0.3*x* (*R* ^2^ = 12%).

**Figure 2:**
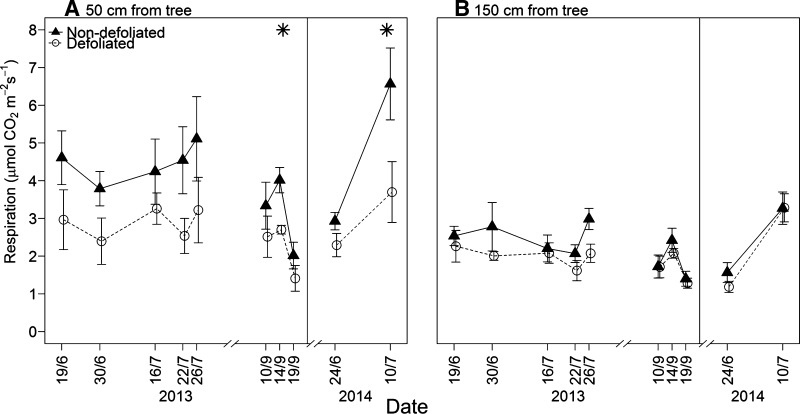
Soil respiration measured over the growing season of 2013 and 2014 at non-defoliated (*closed triangles*) and defoliated (*open circles*) plots at **A** 50 cm and **B** 150 cm from study trees. *Error bars* represent ± one standard error of the mean (*n* = 5). * signifies a significant (*P* < 0.05) effect of defoliation within the statistical model in that year and distance from the tree, according to one degree of freedom Wald tests. Results of a factorial ANOVA test on the whole dataset are shown in Table 1.

Defoliated plots had a significantly lower soil respiration rate (2.63 *μ*mol CO_2_ m^−2^ s^−1^) than non-defoliated plots (3.96 *μ*mol CO_2_ m^−2^ s^−1^) in 2013 when measured 50 cm from the tree (*P* = 0.015; Table 1; Figure 2). However, the effect of defoliation overall was not significant (*P* = 0.068) due to a non-significant response to defoliation at 150 cm from the tree. Overall, there was a significant (*P* = 0.009) effect of distance on respiration rates and no significant interaction (*P* = 0.24) between distance and defoliation. The same pattern, with lower respiration at 50 cm from defoliated trees, continued into 2014.

**Table 1:** Analysis of Variance of Defoliation, Distance from the Base of the Tree, and Their Interaction Effects on Soil Respiration

Data	*y* transformation	Factor	d.f.	*F*	*P*
2013 Respiration	Square root	Distance from tree	**1,8**	**11.68**	**0.009**
		Defoliation	1,8	4.46	0.068
		Distance*Defoliation	1,8	1.62	0.24
2014 Respiration	Square root	Distance from tree	**1,8**	**8.83**	**0.017**
		Defoliation	1,8	3.25	0.11
		Distance*Defoliation	1,8	1.65	0.24

Statistical results correspond to data shown in Figure 2..

Significant (*P* < 0.05) factors are highlighted in bold.

**Figure 3:**
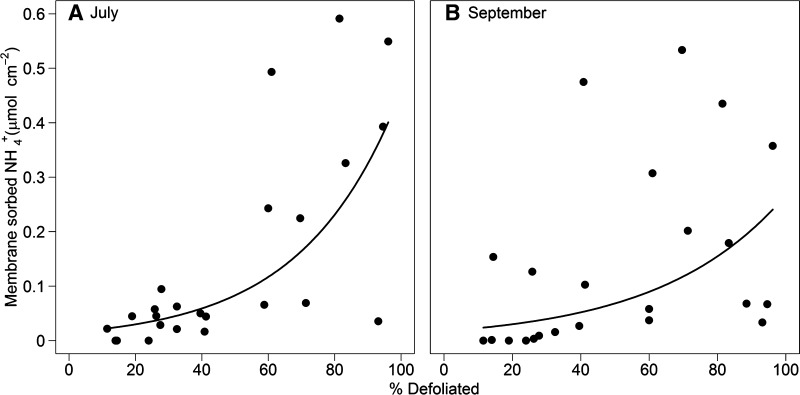
Resin membrane-sorbed ammonium (*µ*mol NH_4_^+^ cm^−2^ membrane) at *LS* plots in **A** July and **B** September in relation to defoliation extent of *B.* *pubescens* (% defoliated). July: $y \, = e^{0.034x \, - \, 4.19}$, *R* ^2^ = 0.56. Non-linear regression: d.f. = 1,22, *t* = 5.49, *P* < 0.001. September: $y \, = e^{0.027x \, - \, 4.05}$ *R* ^2^ = 0.31. Non-linear regression: d.f. = 1,21, *t* = 2.43, *P* = 0.0036).

**Figure 4:**
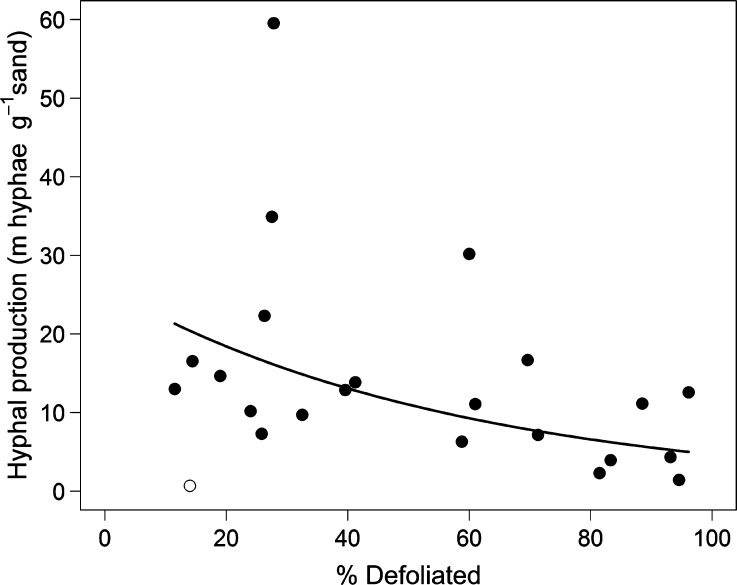
Hyphal ingrowth in relation to defoliation of *B.* *pubescens* (% defoliated) at the *LS* plots. *Line* represents $y \, = e^{ - 0.017x \, + \, 3.26}$, *R* ^2^ = 0.30, without statistical outlier. Non-linear regression: d.f. = 1,20, *t* = 3.17, *P* = 0.0048. Statistical outlier is identified as an *open circle*; no other points were statistical outliers.

There was a significant positive relationship between defoliation extent (% defoliated) and the amount of NH_4_^+^ sorbed to resin membranes during both sampling periods (July and September, 2013). The relationship was strongest in July (*P* < 0.001, Figure 3A); however, it was still present in September (*P* = 0.004, Figure 3B). There was also a significant negative relationship between defoliation extent and EMF hyphal production over the months of June–September (*P* = 0.005, Figure 4). Nitrate was present in very low, almost undetectable, levels, and there was no significant relationship between amount of defoliation and Log_*e*_ + 1 transformed, membrane-sorbed nitrate in either July (*P* = 0.76, *R*_2_ = 0.04%) or September (*P* = 0.69, *R*_2_ = 0.04%). Nitrate data are presented in Supplementary Information, Figures S1 and S2.

**Figure 5:**
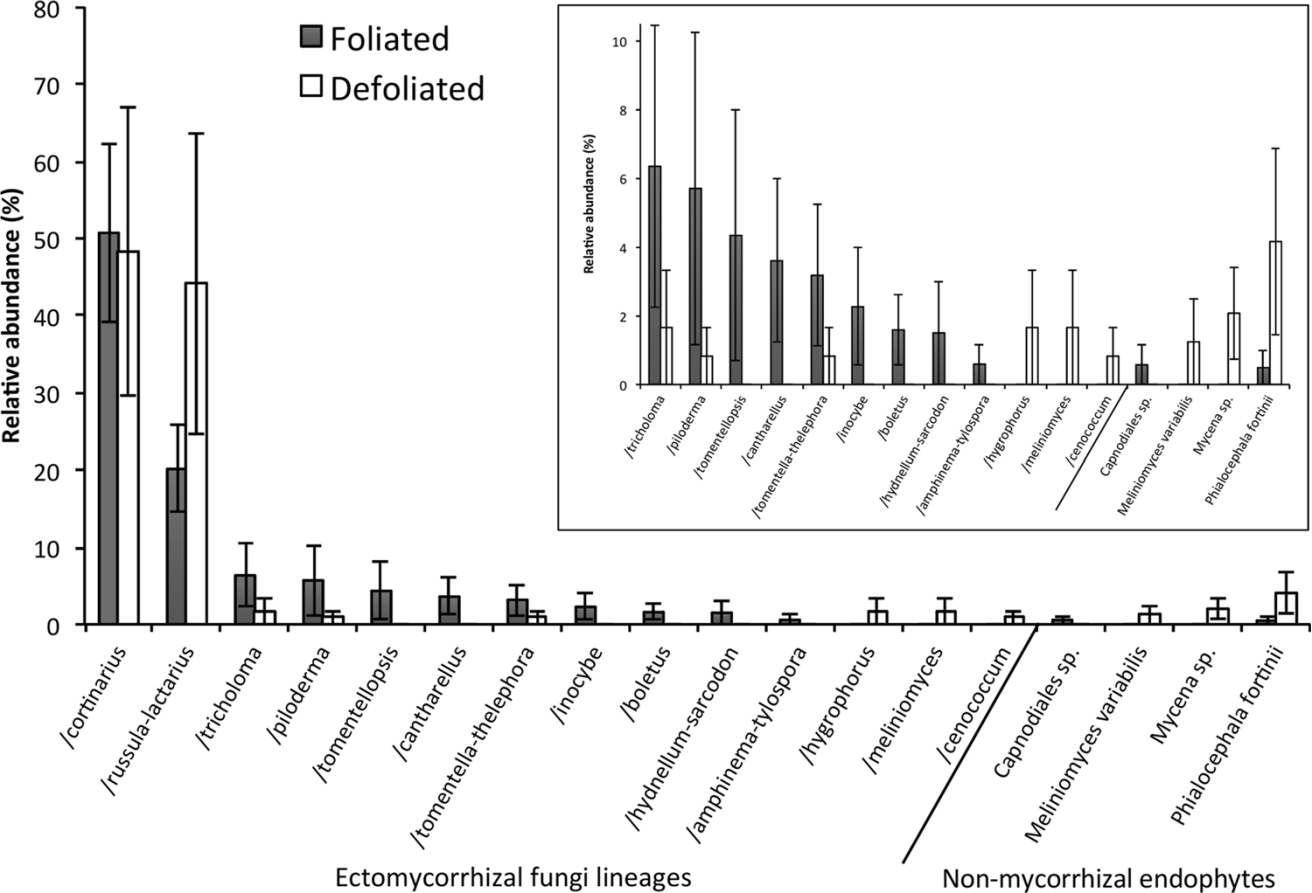
*Main* Relative abundance of ectomycorrhizal (EMF) fungal lineages and non-mycorrhizal (NM) fungi sequenced from ectomycorrhizal root tips of non-defoliated (*grey bars*) and defoliated (*white bars*) *Betula*. *Error bars* are standard error of the mean (*n* = 5) and undefined for unreplicated fungal lineages. Absence in replicates was treated as zero for mean relative abundance calculation. Relative abundance of non-ectomycorrhizal fungi is shown but excluded from analysis. *Inset* relative abundance of all but /cortinarius and /russula-lactarius lineages expressed on 0–12 scale.

The EMF fungal communities present on root tips were significantly affected by defoliation. EMF fungal richness, evenness and diversity declined in association with defoliation (Table 2). Fungal community composition was altered, with some EMF taxa increasing and others decreasing with defoliation. The /cortinarius lineage was the most abundant EMF taxon of both non-defoliated and defoliated trees, representing 48–51% of relative EMF abundance (Figure 5), and its relative abundance was not affected by defoliation. In contrast, defoliation significantly affected the relative abundance of the /russula-lactarius lineage, which increased from 20% to 44% in non-defoliated and defoliated trees, respectively. Additionally, the EMF lineages /tomentella-thelephora, /tomentellopsis, /piloderma, /cantharellus, /inocybe, /hydnellum-sarcodon, /amphinema-tylospora and /boletus, which together accounted for about 30% of EMF fungi of non-defoliated trees, collectively declined to 3% of the EMF community of defoliated trees. The lineages /hygrophorus, /meliniomyces and /cenococcum were present as EMF symbionts of roots obtained from single defoliated plots and were not detected in non-defoliated plots. Non-mycorrhizal fungi most closely related to root endophytes that included *Phialocephala fortinii*, *P.* *sphaeroides* and *Meliniomyces variabilis*, and free-living litter decomposers (two *Mycena* spp.) were recovered mainly from EMF roots of defoliated trees (Table S1).

**Table 2:** Effect of Defoliation on Ectomycorrhizal Fungal Community Richness (*S*), Evenness (*E*), Shannon Diversity (*H*′), Simpson Diversity (*D*′) and Composition

	*S*	*E*	*H*′	*D*′	Composition
Non-defoliated	4.8 ± 1.4	0.83 ± 0.07	1.2 ± 0.31	0.60 ± 0.12	
Defoliated	2.6 ± 0.9	0.43 ± 0.19	0.5 ± 0.24	0.28 ± 0.13	
*P* > |*t*|	0.04^a^	0.05^a^	0.04^a^	0.05^a^	0.04^b^

Values are means ± standard error (*n* = 5).

^a^Paired t-test.

^b^Blocked multi-response permutation procedure *t* statistic.

**Figure 6:**
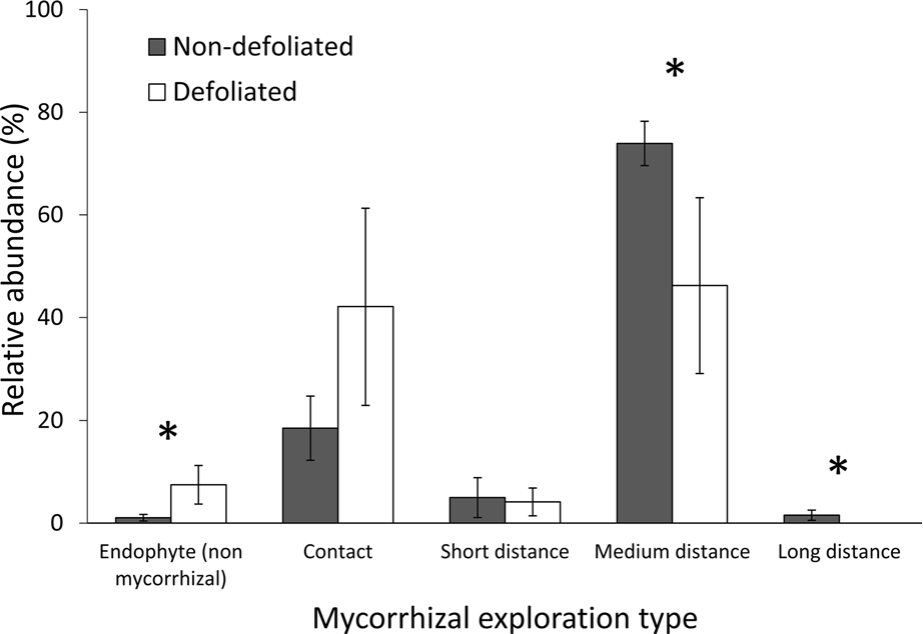
Effect of defoliation (non-defoliated in *white* defoliated in *black*), on relative abundance of non-mycorrhizal fungi and exploration types (ETs) of ectomycorrhizal fungi. Absence within replicates was counted as zero for mean relative abundance calculation. *Bars* are ± one standard error of the mean. *Asterisks* indicate defoliation affected relative abundance of an ET (Holm–Tukey test) at *P* < 0.05.

Amongst EMF lineages, those with high extraradical mycelium biomass (medium- and long-distance soil exploration strategy (Hobbie and Agerer 2010)) declined in relative abundance, from colonising 76% of EMF roots of non-defoliated trees to 43% of EMF roots of defoliated trees (Figure 5). This decline of high-biomass EMF ET with defoliation coincided with increased relative abundance of contact ET (consisting of /russula-lactarius) and of non-mycorrhizal fungi (Figures 5 and 6).

## Discussion

This study documents a series of changes in soil processes in response to defoliation that, taken together, suggest a slowdown of biogeochemical cycling. The data suggest that a reduction in ectomycorrhizal (EMF) production and a shift in EMF community composition could be important contributory factors involved in the slowdown of C and N cycling in the soil.

Previous work in this system has shown that large defoliation events drastically reduce the strength of the ecosystem C sink (Heliasz and others 2011). Here we demonstrate that the reduction in C assimilation also slows the loss of C from the soil (as respiration). This was only statistically significant, however, closer to the tree base (at 50 cm), presumably where the tree has a greater influence on soil carbon cycling rates. These data suggest that the belowground respiration rate is sensitive to a reduction in aboveground C assimilation, as observed in experimental girdling and trenching experiments (Högberg and others 2001; Brzostek and others 2015). The effect of the defoliation on C cycling across the rest of the forest (≥150 cm from tree base) may be negligible, as we observed no reduction in respiration further away from study trees. There will therefore be large areas of forest soil where soil CO_2_ efflux may not be directly affected by the defoliation of the canopy, although subtler (undetected here) effects cannot be ruled-out, and could relate, for example, to changes in soil thermal and/or moisture regimes in response to defoliation.

This study in forest plots which have undergone almost complete defoliation in a relatively unproductive ecosystem (Myneni and others 2001; Karlsen and others 2008) shows the opposite response to more productive ecosystems, which experienced a smaller reduction in belowground C allocation. In a temperate deciduous system, belowground respiration rates increased in response to relatively mild defoliation (8% less foliage than control; (Frost and Hunter 2004)). This was similar to patterns of increased C allocation belowground by plants in response to partial herbivory (Bardgett and Wardle 2003; Orians and others 2011): Here the authors explained the increase in C flux as being due to a combination of increased root growth, turnover, activity and labile C input, all as mechanisms to recover N that was lost from leaf biomass (Frost and Hunter 2004). All of these processes depend on a high flux of autotrophic C to maintain roots and associated mycorrhizal symbionts (Litton and others 2007; Ekblad and others 2013; Brzostek and others 2015); something that was not possible in the present study as the defoliated trees suffered almost complete defoliation. In a subalpine forest suffering a bark beetle outbreak, as productivity decreased with tree death, ecosystem respiration decreased, signifying an overall slowdown of the forest C cycle (Moore and others 2013). This is analogous to the present study system; soil carbon cycling in sub-arctic forests relies on autotrophic C supply to stimulate the activity of microbial communities and continue the decomposition of soil organic matter (Hartley and others 2012). Our study demonstrates that when this C supply is cut, cycling in the soil slows, further supporting the hypothesis that it is recently fixed carbon that drives this cycle (Hartley and others 2012).

Free nitrogen (NH_4_^+^) in the soil was increased by an order of magnitude in extreme cases of defoliation compared to relatively unaffected areas of forest. Furthermore, free NH_4_^+^–N in the soil increased exponentially with defoliation extent, implying that accumulation of N in the soil results from multiple processes as opposed to simply being a linear function of frass input or any other single factor. It is therefore clear that the N cycle in this system was drastically altered by defoliation. One important likely change in N flux with defoliation was a reduction in root uptake (Kosola and others 2001). Additionally, although not measured directly, it is reasonable to assume that insect frass input scaled linearly with the severity of defoliation in the forest (Lovett and Ruesink 1995; Lovett and others 2002). Therefore, the large amount of free N observed in the soil was probably a product of increased direct N input via frass, as well as on-going mineralisation (Sjögersten and Wookey 2005), which was not balanced by an increase in uptake by roots and microbial biomass (Lovett and Ruesink 1995). In fact, root growth and uptake of N were likely dramatically reduced in the severely defoliated plots (Kosola and others 2001; Cigan and others 2015; Saravesi and others 2015). In this case, the argument for reduced root activity due to reductions in autotrophic C supply is also supported by the observed reduction in soil respiration rates, similar to what is observed when trees are girdled (Högberg and others 2001). In contrast to NH_4_^+^–N, exchangeable NO_3_^−^–N was present at very low levels and was not related to the extent of defoliation of the forest canopy (Figures S1 and S2). Although not addressing defoliation effects, previous work by Sjögersten and Wookey (2005) identified a similar predominance (~2 orders of magnitude) of NH_4_^+^–N over NO_3_^−^–N in a nearby mountain birch forest. Furthermore, isotope tracing studies have also shown that available NO_3_^−^–N is significantly lower than NH_4_^+^–N in the soil, and is relatively unresponsive to defoliation events (Christenson and others 2002).

Work at a different defoliated sub-arctic birch forest in Finland also showed that free N availability increased in defoliated plots, with the authors of this study suggesting that defoliation shifts the decomposer community to one in which bacteria become more dominant, while the fungal decomposition pathway is weakened (Kaukonen and others 2013). Therefore we speculate that N immobilisation by bacteria may have increased in the current study as their growth can be stimulated by the addition of free N when in combination with equally accessible C inputs (Bååth and others 1978), as is the case with defoliation events (Lovett and Ruesink 1995).

We documented a decline in EMF hyphal production with increasing defoliation which was likely caused directly by a reduction in C supply from the defoliated host trees (Gehring and Whitham 1991; Gehring and others 1997; Kuikka and others 2003)). This decline in hyphal production was concomitant with a shift in the composition of the remaining EMF community. This has previously been observed in the soil after defoliation (Saravesi and others 2015) and in the present study, on root tips. Growth of EMF mycelium is known to be most directly influenced by the amount of carbon that is made available by autotrophic hosts (Ekblad and others 2013; Wallander and Ekblad 2015). Experimental reduction of this autotrophic C supply leads to loss of EMF biomass in the soil (Högberg and others 2001). Linked to C fluxes, EMF fungi are also an important sink for N in boreal forests (Mikusinska and others 2013; Näsholm and others 2013b) and can contain up to 200 kg N ha^−1^ in their biomass (Wallander and others 2004). It has been suggested that EMF fungi immobilise more N, and transfer less to trees, in low N environments in order to maintain their host’s reliance on them (Näsholm and others 2013b). Therefore, a reduction in EMF hyphal production, as was observed here, may have contributed to the flush of inorganic N as the EMF failed to immobilise the excess N from the larval frass input (Lovett and Ruesink 1995).

The shift in community composition of the remaining EMF root tips in defoliated plots may also have had a significant role in the reduced immobilisation of N. There was a shift in the EMF fungi colonising *B.* *pubescens* roots from medium and long-distance ET in non-defoliated plots to smooth-mantled contact lineages such as /russula-lactarius in the defoliated plots (Agerer and others 2012). This is consistent with the hypothesis that when a host tree is defoliated, EMF species with a lower C requirement hold a competitive advantage over those that invest in more extensive soil exploration (Saikkonen and others 1999; Markkola and others 2004). A similar shift to increased dominance of the /russula-lactarius lineage on EMF root tips and a reduction of mycelial biomass on EMF roots was observed in another mountain birch forest in response to defoliation (Saravesi and others 2015). The opposite occurred in a warming experiment on the Alaskan tundra where the biomass of *B.* *nana* increased; there, a shift to the more explorative *Cortinarius* spp. from the lower plant C investment *Russula* spp., was observed, presumably as a result of increased plant C supplied belowground (Deslippe and others 2011). In this study, *Cortinarius* spp. remained unchanged in relative abundance, which could be of ecological significance; *Cortinarius* spp. are thought to be of particular importance to N cycling in arctic and boreal ecosystems because they use oxidative enzymes to extract N from complex organic molecules (Bödeker and others 2014) and could be important species in the transfer of N to host plants (Deslippe and others 2015). Species of *Cortinarius* have been shown to reduce expression of genes related to oxidative enzyme production under N fertilisation (Bödeker and others 2014). Here, where direct uptake of excess N under defoliated conditions would seem more favourable, *Cortinarius* spp. may have also reduced use of enzymes, which may slow decomposition of soil organic carbon. In our study, the shift in the composition of the EMF community likely contributed to the reduction of EMF hyphal production that was observed in highly defoliated plots and may also have indirectly contributed towards high concentrations of free NH_4_^+^–N in the soil.

An intriguing change in fungal community composition on root tips observed here was a clear increase in non-mycorrhizal endophytes. Although these fungi are common across the world, especially in stressful environments, their ecological function is largely unknown (Rodriguez and others 2009). One suggestion is that they become preferred as a low C cost symbiont when a plant is under stress in order to protect roots from pathogens (Mandyam and Jumpponen 2005), which is a reasonable explanation in this case. Alternatively, evidence has suggested that root endophytes can increase on roots in response to defoliation and that this may be in order to take advantage of root die-back (Saravesi and others 2014). Further work addressing why root endophytes increase beneath defoliated forests is clearly an interesting avenue of research and may shed further light on this poorly understood group of fungi.

Moth outbreaks in this area are known to be limited by minimum winter temperature, with a temperature lower than around—35 °C known to freeze and kill over-wintering eggs (Tenow and Nilssen 1990). Increases in temperature and concurrent reductions in the number of days below—35 °C have been shown to increase the range of both *E.* *autumnata* and *O.* *brumata* (Jepsen and others 2008). The latter has undergone particularly large increases in its range as it is more sensitive to cold temperatures than *E.* *autumnata*. In this study, there was an observed, although weak relationship between the density of trees and severity of defoliation. This was likely due to conditions that determine insect survival over winter through the differential probability of lethal super-cooling of eggs (Tenow and Nilssen 1990). At a larger scale, lethal winter air temperatures can be influenced by topography (Tenow and Nilssen 1990). At smaller scales, the availability of over-wintering sites can be the critical control of caterpillar survival and resulting defoliation (Bylund 1997). This was evident in old forests that had high outbreak numbers (many stems and over-wintering sites) and young forest with the opposite (Bylund 1997). Relationships such as these may be useful, in combination with ecosystem studies such as this and others (Heliasz and others 2011; Kaukonen and others 2013; Saravesi and others 2015), to derive a better understanding of the larger scale influence of Winter and Autumn Moths on whole ecosystem processes.

Herbivorous insect distributions and populations are known to be particularly responsive to winter and summer temperature increases (Bale and others 2002) and with this in mind, along with the observed past changes in moth ranges in relation to warming (Jepsen and others 2008), it appears likely that this kind of disturbance will increase in severity and magnitude in the coming century (Bale and others 2002) and may also have the potential to modulate forest expansion.

In summary, a large-scale defoliation event by *O.* *brumata* and *E.* *autumnata* caused a number of cascading effects in sub-arctic mountain birch forests. A reduced delivery of autotrophic C to the rhizosphere may have contributed towards accumulation of mineral N in the soil, which may have been linked in part to the altered composition and growth of the EMF community. In defoliation events, the accumulation of free N in the soil is initiated by frass inputs (Lovett and others 2002), but this phenomenon may persist in this study ecosystem because the influence of trees in the soil is diminished to the point where they can no longer drive immobilisation, either by direct uptake (Russell and others 2004) or through EMF fungi (Näsholm and others 2013b). We argue that the defoliated mountain birch forest resembles an N-saturated ecosystem (Aber 1992) where biological immobilisation of N cannot keep pace with inputs due to a reduction in ‘top-down’ control. A research priority remains to measure further the longevity of response observed here in order to understand better the stoichiometry of C and N in the wake of these important disturbances. The EMF community is known to be a key link between changes in autotrophic C supply and cycling of C and N in the soil. Defoliation events are a feature of many forest ecosystems (particularly in sub-arctic, boreal and temperate regions), yet we still know little of the complex processes and cascading interactions that they drive. In an era of rapid environmental changes, where short-lived and mobile insect species are able to respond rapidly to new opportunities for range expansion, the severity of ‘outbreak’ years will likely have more profound ecosystem impacts. Further work needs to focus on the interactions and potentially non-linear effects of defoliation events to understand better how these important ecological events exert control over ecosystem processes and the C budget of these forests.

## Electronic supplementary material

Below is the link to the electronic supplementary material.

Supplementary material 1 (DOCX 182 kb)
