# Supplementary material for: Slowed Biogeochemical Cycling in Sub-arctic Birch Forest Linked to Reduced Mycorrhizal Growth and Community Change after a Defoliation Event
Source: Ecosystems. 2016 Aug 25;20(2):316–30. doi: 10.1007/s10021-016-0026-7 (PMC7089692; doi:10.1007/s10021-016-0026-7)
Supplement: Supplementary file 1 — Supplementary material 1 (DOCX 182 kb) [file 10021_2016_26_MOESM1_ESM.docx]

Supplemental Information for ‘Slowed biogeochemical cycling in sub-arctic birch forest linked to reduced mycorrhizal growth and community change after a defoliation event’.

Authors: Thomas C. Parker, Jesse Sadowsky, Haley Dunleavy, Jens-Arne Subke, Serita Frey & Philip A. Wookey


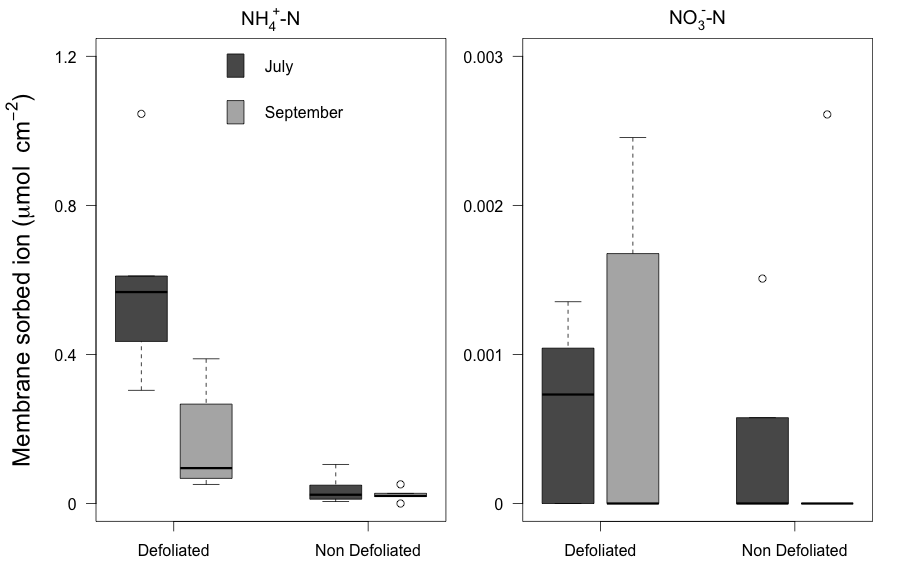


**Figure S1:** Resin membrane-sorbed ammonium (left) and nitrate (right) at defoliated and non defoliated trees in July (dark grey) and September (light grey). Boxes represent inter-quartile range (IQR) with the median value of the data, whiskers signify the normal range of the data and open circles represent any outlying points (n = 5).


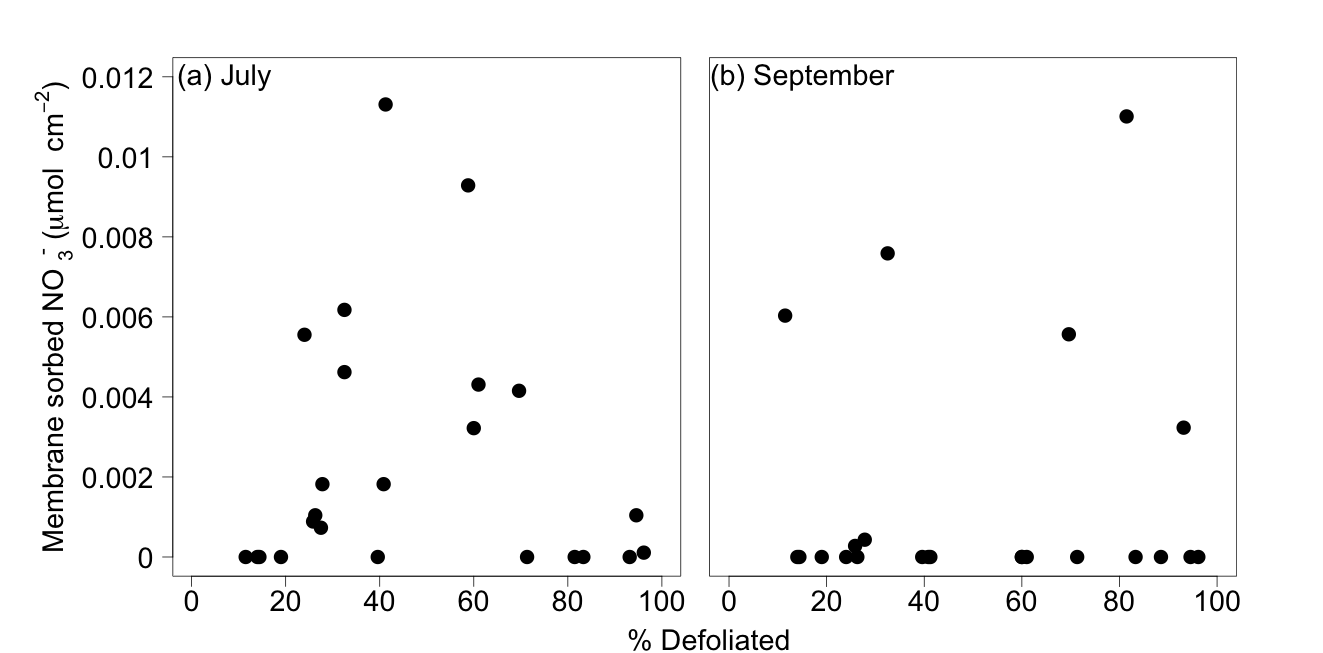


**Figure S2:** Resin membrane-sorbed nitrate (µmol NO_3_^-^ cm^-2^ membrane) at *LS* plots in (a) July and (b) September in relation to defoliation extent of *B. pubescens* (% defoliated). There is no significant relationship between amount of defoliation and Log_e_ +1 transformed, membrane sorbed nitrate in either July (*P* = 0.76, R_2_ =0.04%) or September (*P* = 0.69, R_2_ =0.04%).

**Table S1.** Reference sequence annotation, relative abundance, and frequency of occurrence of ectomycorrhizal (ECM) fungi in non-defoliated and defoliated *Betula* forests in Abisko, Sweden. Values for genus- or higher-level taxonomic groups are shaded.

| Reference taxon | | | |  | NCBI/UNITE Accession | | Match (%) | | UNITE Species Hypothesis | | Mycorrhizal Exploration Type^#^ | | Non-defoliated Defoliated  ^†^ ^†^ *ƒ*^‡^ *ƒ* (%) ( /5) (%) ( /5) | | | | | | | | | | | | | | | |  |
| --- | --- | --- | --- | --- | --- | --- | --- | --- | --- | --- | --- | --- | --- | --- | --- | --- | --- | --- | --- | --- | --- | --- | --- | --- | --- | --- | --- | --- | --- |
| ECTOMYCORRHIZAL FUNGI | | | | | | | | | | | | | | | | | | | | | | | | | | | | |  |
| Basidiomycota | | | |  |  | |  | |  | |  | |  | | | | | | | | | | | | | | | |  |
| Agaricales | | | |  |  | |  | |  | |  | |  | | | | | | | | | | | | | | | |  |
| Cortinariaceae | | | |  |  | |  | |  | |  | |  | | | | | | | | | | | | | | | |  |
|  | | /cortinarius | | |  | |  | |  | | MDF | | 50 | | ± | | 12 | | 5/5 | | 55 | | ± | | 18 | | 4/5 | |  |
|  | | *Cortinarius alboviolaceus* | | | JQ724019.1 | | 99 | | SH221787.06FU | | MDF | | 38 | | ± | | 13 | | 2/5 | |  | |  | |  | |  | |  |
|  | | *Cortinarius armillatus* | | | DQ114744.1 | | 99 | | SH221779.06FU | | MDF | |  | |  | |  | |  | | 21 | |  | |  | | 1/5 | |  |
|  | | *Cortinarius atrocoeruleus* | | | JQ724019.1 | | 99 | | SH232829.06FU | | MDF | | 12 | |  | |  | | 1/5 | |  | |  | |  | |  | |  |
|  | | *Cortinarius balaustinus* | | | AF389153.1 | | 98 | | SH220396.06FU | | MDF | | 25 | |  | |  | | 1/5 | | 31 | |  | |  | | 1/5 | |  |
|  | | *Cortinarius caperatus* | | | FJ845425.1 | | 99 | | SH191824.06FU | | MDF | | 6 | |  | |  | | 1/5 | | 41 | | ± | | 20 | | 3/5 | |  |
|  | | *Cortinarius casimiri* | | | HQ604710.1 | | 99 | | SH232830.06FU | | MDF | | 3 | | ± | | 0.2 | | 2/5 | | 33 | |  | |  | | 1/5 | |  |
|  | | *Cortinarius cedriolens* | | | FJ552786.1 | | 99 | | SH232841.06FU | | MDF | | 7 | |  | |  | | 1/5 | |  | |  | |  | |  | |  |
|  | | *Cortinarius collinitus* | | | DQ367896.1 | | 99 | | SH191855.06FU | | MDF | | 5 | |  | |  | | 1/5 | |  | |  | |  | |  | |  |
|  | | *Cortinarius delibutus* | | | UDB002173 | | 99 | | SH220385.06FU | | MDF | | 8 | | ± | | 3 | | 2/5 | |  | |  | |  | |  | |  |
|  | | *Cortinarius laetus* | | | AF389170.1 | | 99 | | SH192000.06FU | | MDF | |  | |  | |  | |  | | 4 | |  | |  | | 1/5 | |  |
|  | | *Cortinarius* sp. | | | EU597035.1 | | 96 | | SH191963.06FU | | MDF | | 5 | |  | |  | | 1/5 | |  | |  | |  | |  | |  |
|  | | *Cortinarius* sp. | | | FJ039656.1 | | 99 | | SH192083.06FU | | MDF | |  | |  | |  | |  | | 7 | |  | |  | | 1/5 | |  |
|  | | *Cortinarius* sp. | | | JN032537.1 | | 99 | | SH191806.06FU | | MDF | | 10 | | ± | | 4 | | 2/5 | |  | |  | |  | |  | |  |
|  | | *Cortinarius* sp. | | | UDB002209 | | 99 | | SH191882.06FU | | MDF | | 38 | | ± | | 22 | | 2/5 | |  | |  | |  | |  | |  |
| Hygrophoraceae | | | |  |  | |  | |  | |  | |  | |  | |  | |  | |  | |  | |  | |  | |  |
|  | | /hygrophorus | | |  | |  | |  | | SD | |  | |  | |  | |  | | 7 | |  | |  | | 1/5 | |  |
|  | | *Hygrophorus pudorinus* | | | FJ845408.1 | | 88 | | SH024898.06FU | | SD | |  | |  | |  | |  | | 7 | |  | |  | | 1/5 | |  |
| Inocybaceae | | | |  |  | |  | |  | |  | |  | |  | |  | |  | |  | |  | |  | |  | |  |
|  | | /inocybe | | |  | |  | |  | | SD | | 6 | | ± | | 3 | | 2/5 | |  | |  | |  | |  | |  |
|  | | *Inocybe obscurobadia* | | | AM882802.2 | | 97 | | SH001193.06FU | | SD | | 2 | |  | |  | | 1/5 | |  | |  | |  | |  | |  |
|  | | *Inocybe petiginosa* | | | EF218781.1 | | 99 | | SH208978.06FU | | SD | | 9 | |  | |  | | 1/5 | |  | |  | |  | |  | |  |
| Tricholomataceae | | | |  |  | |  | |  | |  | |  | |  | |  | |  | |  | |  | |  | |  | |  |
|  | | /tricholoma | | |  | |  | |  | | MDF | | 15 | | ± | | 4 | | 2/5 | | 7 | |  | |  | | 1/5 | |  |
|  | | *Tricholoma album* | | | UDB002398 | | 99 | | SH194000.06FU | | MDF | |  | |  | |  | |  | | 4 | |  | |  | | 1/5 | |  |
|  | | *Tricholoma flavovirens* | | | AF349689.1 | | 99 | | SH192989.06FU | | MDF | | 13 | | ± | | 1 | | 2/5 | | 4 | |  | |  | | 1/5 | |  |
|  | | *Tricholoma virgatum* | | | UDB011594 | | 100 | | SH194223.06FU | | MDF | | 6 | |  | |  | | 1/5 | |  | |  | |  | |  | |  |
| Atheliales | | | |  |  | |  | |  | |  | |  | |  | |  | |  | |  | |  | |  | |  | |  |
| Atheliaceae | | | |  |  | |  | |  | |  | |  | |  | |  | |  | |  | |  | |  | |  | |  |
|  | | /amphinema-tylospora | | |  | |  | |  | | SD | | 3 | |  | |  | | 1/5 | |  | |  | |  | |  | |  |
|  | | *Tylospora* sp. | | | HM189733.1 | | 100 | | SH229868.06FU | | SD | | 3 | |  | |  | | 1/5 | |  | |  | |  | |  | |  |
|  | | /piloderma | | |  | |  | |  | | MDF | | 14 | | ± | | 9 | | 2/5 | | 4 | |  | |  | | 1/5 | |  |
|  | | *Piloderma olivaceum* | | | JQ711859.1 | | 98 | | SH212379.06FU | | MDF | | 12 | | ± | | 8 | | 2/5 | | 4 | |  | |  | | 1/5 | |  |
|  | | *Piloderma* sp. | | | JQ711935.1 | | 99 | | SH212383.06FU | | MDF | | 3 | |  | |  | | 1/5 | |  | |  | |  | |  | |  |
| Boletales | | | |  |  | |  | |  | |  | |  | |  | |  | |  | |  | |  | |  | |  | |  |
| Boletaceae | | | |  |  | |  | |  | |  | |  | |  | |  | |  | |  | |  | |  | |  | |  |
|  | | /boletus | | |  | |  | |  | | LD | | 4 | | ± | | 1 | | 2/5 | |  | |  | |  | |  | |  |
|  | | *Leccinum scabrum* | | | UDB001608 | | 99 | | SH197538.06FU | | LD | | 4 | | ± | | 1 | | 2/5 | |  | |  | |  | |  | |  |
| Cantharellales | | | |  |  | |  | |  | |  | |  | |  | |  | |  | |  | |  | |  | |  | |  |
| Hydnaceae | | | |  |  | |  | |  | |  | |  | |  | |  | |  | |  | |  | |  | |  | |  |
|  | | /cantharellus | | |  | |  | |  | | MDF, MDS | | 9 | | ± | | 3 | | 2/5 | |  | |  | |  | |  | |  |
|  | | *Hydnum umbilicatum* | | | AJ547885.1 | | 97 | | SH214526.06FU | | MDS | | 11 | |  | |  | | 1/5 | |  | |  | |  | |  | |  |
|  | | *Sistotrema* sp. | | | FN669254.1 | | 99 | | SH219329.06FU | | MDF | | 6 | |  | |  | | 1/5 | |  | |  | |  | |  | |  |
| Russulales | | | |  |  | |  | |  | |  | |  | |  | |  | |  | |  | |  | |  | |  | |  |
| Russulaceae | | | |  |  | |  | |  | |  | |  | |  | |  | |  | |  | |  | |  | |  | |  |
|  | | /russula-lactarius | | |  | |  | |  | | C, SD | | 20 | | ± | | 6 | | 5/5 | | 70 | | ± | | 15 | | 3/5 | |  |
|  | | | *Lactarius* | | |  | |  | |  | | C | | 8 | | ± | | 3 | | 3/5 | | 83 | | ± | | 17 | | 2/5 | |
|  | | *Lactarius pilatii* | | | UDB018157 | | 99 | | SH238107.06FU | | C | | 5 | | ± | | 2 | | 2/5 | |  | |  | |  | |  | |  |
|  | | *Lactarius rufus* | | | KF241543.1 | | 99 | | SH191391.06FU | | C | |  | |  | |  | |  | | 67 | |  | |  | | 1/5 | |  |
|  | | *Lactarius tabidus* | | | HM189825.1 | | 99 | | SH193869.06FU | | C | |  | |  | |  | |  | | 8 | |  | |  | | 1/5 | |  |
|  | | *Lactarius trivialis* | | | UDB000365 | | 99 | | SH238110.06FU | | C | | 13 | |  | |  | | 1/5 | | 92 | |  | |  | | 1/5 | |  |
|  | | | *Russula* | | |  | |  | |  | | C, SD | | 15 | | ± | | 5 | | 4/5 | | 48 | |  | |  | | 1/5 | |
|  | | *Russula gracillima* | | | KF002779.1 | | 99 | | SH224403.06FU | | C | | 7 | |  | |  | | 1/5 | |  | |  | |  | |  | |  |
|  | | *Russula nuoljae* | | | UDB002530 | | 99 | | SH207687.06FU | | C | | 12 | | ± | | 6 | | 4/5 | | 48 | |  | |  | | 1/5 | |  |
|  | | *Russula versicolor* | | | UDB001641 | | 99 | | SH224391.06FU | | SD | | 3 | |  | |  | | 1/5 | |  | |  | |  | |  | |  |
| Thelephorales | | | |  |  | |  | |  | |  | |  | |  | |  | |  | |  | |  | |  | |  | |  |
| Bankeraceae | | | |  |  | |  | |  | |  | |  | |  | |  | |  | |  | |  | |  | |  | |  |
|  | | /hynellum-sarcodon | | |  | |  | |  | | MDM | | 7 | |  | |  | | 1/5 | |  | |  | |  | |  | |  |
|  | | *Sarcodon* sp. | | | UDB015699 | | 100 | | SH227933.06FU | | MDM | | 7 | |  | |  | | 1/5 | |  | |  | |  | |  | |  |
|  | Thelephoraceae | | |  |  | |  | |  | | SD, MDS | | 13 | | ± | | 3 | | 3/5 | | 4 | |  | |  | | 1/5 | |  |
|  | | /tomentella-thelephora | | |  | |  | |  | | SD, MDS | | 8 | | ± | | 2 | | 2/5 | | 4 | |  | |  | | 1/5 | |  |
|  | | Thelephoraceae sp. | | | U83467.1 | | 99 | | SH195967.06FU | | MDS | | 7 | |  | |  | | 1/5 | |  | |  | |  | |  | |  |
|  | | *Tomentella lapida* | | | U83480.1 | | 98 | | SH199020.06FU | | SD | | 6 | |  | |  | | 1/5 | |  | |  | |  | |  | |  |
|  | | *Tomentella* sp. | | | FJ553031.1 | | 99 | | SH219847.06FU | | SD | | 2 | |  | |  | | 1/5 | | 4 | |  | |  | | 1/5 | |  |
|  | | /tomentellopsis | | |  | |  | |  | | MDF/MDS | | 11 | | ± | | 8 | | 2/5 | |  | |  | |  | |  | |  |
|  | | *Tomentellopsis* sp. | | | UDB018589 | | 99 | | SH199526.06FU | | MDF/MDS | | 11 | | ± | | 8 | | 2/5 | |  | |  | |  | |  | |  |
| Ascomycota | | | |  |  | |  | |  | |  | |  | |  | |  | |  | |  | |  | |  | |  | |  |
| Mytilinidiales | | | |  |  | |  | |  | |  | |  | |  | |  | |  | |  | |  | |  | |  | |  |
| Gloniaceae | | | |  |  | |  | |  | |  | |  | |  | |  | |  | |  | |  | |  | |  | |  |
|  | | /cenococcum | | |  | |  | |  | | SD | |  | |  | |  | |  | | 4 | |  | |  | | 1/5 | |  |
|  | | *Cenococcum geophilum* | | | JN943891.1 | | 99 | | SH196545.06FU | | SD | |  | |  | |  | |  | | 4 | |  | |  | | 1/5 | |  |
| Helotiales | | | |  |  | |  | |  | |  | |  | |  | |  | |  | |  | |  | |  | |  | |  |
| Incertae sedis | | | |  |  | |  | |  | |  | |  | |  | |  | |  | |  | |  | |  | |  | |  |
|  | | /meliniomyces | | |  | |  | |  | | SD | |  | |  | |  | |  | | 7 | |  | |  | | 1/5 | |  |
|  | | *Meliniomyces bicolor* | | | HM164675.1 | | 100 | | SH207165.06FU | | SD | |  | |  | |  | |  | | 7 | |  | |  | | 1/5 | |  |
| NON-ECTOMYCORRHIZAL FUNGI | | | | | | | | | | | | | | | | | | | | | | | | | | | | |  |
| Basidiomycota | | | |  |  | |  | |  | |  | |  | |  | |  | |  | |  | |  | |  | |  | |  |
| Agaricales | | | |  |  | |  | |  | |  | |  | |  | |  | |  | |  | |  | |  | |  | |  |
| Mycenaceae | | | |  |  | |  | |  | |  | |  | |  | |  | |  | |  | |  | |  | |  | |  |
|  | | | *Mycena* | |  | |  | |  | |  | |  | |  | |  | |  | | 5 | | ± | | 1 | | 2/5 | |  |
|  | | *Mycena simia* | | | GU234138.1 | | 99 | | SH237366.06FU | |  | |  | |  | |  | |  | | 4 | |  | |  | | 1/5 | |  |
|  | | *Mycena* sp. | | | HM069358.1 | | 100 | | SH193138.06FU | |  | |  | |  | |  | |  | | 7 | |  | |  | | 1/5 | |  |
| Capnodiales | | | |  |  | |  | |  | |  | |  | |  | |  | |  | |  | |  | |  | |  | |  |
| Incertae sedis | | | |  |  | |  | |  | |  | |  | |  | |  | |  | |  | |  | |  | |  | |  |
|  | | *Toxicocladosporium*  *strelitziae* | | | JX069874.1 | | 98 | | SH196751.06FU | |  | | 3 | |  | |  | | 1/5 | |  | |  | |  | |  | |  |
| Ascomycota | | | |  |  | |  | |  | |  | |  | |  | |  | |  | |  | |  | |  | |  | |  |
| Helotiales | | | |  |  | |  | |  | |  | |  | |  | |  | |  | |  | |  | |  | |  | |  |
| Vibrisseaceae | | | |  |  | |  | |  | |  | |  | |  | |  | |  | |  | |  | |  | |  | |  |
|  | | | *Phialocephala* | |  | |  | |  | |  | | 2 | |  | |  | | 1/5 | | 10 | | ± | | 3 | | 2/5 | |  |
|  | | *Phialocephala fortinii* | | | DQ497924.1 | | 99 | | SH213468.06FU | |  | | 2 | |  | |  | | 1/5 | | 7 | |  | |  | | 1/5 | |  |
|  | | *Phialocephala sphaeroides* | | | JQ711837.1 | | 99 | | SH213470.06FU | |  | |  | |  | |  | |  | | 13 | |  | |  | | 1/5 | |  |
| Incertae sedis | | | |  |  | |  | |  | |  | |  | |  | |  | |  | |  | |  | |  | |  | |  |
| Incertae sedis | | | |  |  | |  | |  | |  | |  | |  | |  | |  | |  | |  | |  | |  | |  |
|  | | *Meliniomyces variabilis* | | | FN565286.1 | | 98 | | SH207164.06FU | |  | |  | |  | |  | |  | | 6 | |  | |  | | 1/5 | |  |

^#^Abbreviations: C, contact; SD, short-distance; MDF, medium-distance fringe; MDM, medium-distance mat; MDS, medium-distance smooth; LD, long-distance. References: Agerer (2006), Tedersoo & Smith (2013), Beenken (2004) , Weigt and others (2012), and Jakucs and others (2015).

^†^ ± : Mean and standard error of taxon relative abundance in transect-replicate samples where it occurred; further detail in *Materials and Methods..*

^‡^*ƒ*: Frequency of occurrence in five transect-replicate samples

**Supplemental information references**

Agerer R. 2006. Fungal relationships and structural identity of their ectomycorrhizae. Mycological Progress 5:67–107. <Go to ISI>://WOS:000240954900001

Jakucs E, Erős-Honti Z, Seress D, Kovács GM. 2015. Enhancing our understanding of anatomical diversity in Tomentella ectomycorrhizas: characterization of six new morphotypes. Mycorrhiza 25:419–29. http://www.ncbi.nlm.nih.gov/pubmed/25564437. Last accessed 12/01/2016

Tedersoo L, Smith ME. 2013. Lineages of ectomycorrhizal fungi revisited: Foraging strategies and novel lineages revealed by sequences from belowground. Fungal Biology Reviews 27:83–99. <Go to ISI>://WOS:000328802000002

Weigt RB, Raidl S, Verma R, Agerer R. 2012. Exploration type-specific standard values of extramatrical mycelium - a step towards quantifying ectomycorrhizal space occupation and biomass in natural soil. Mycological Progress 11:287–97. <Go to ISI>://WOS:000300079600027
